# Relationship Between the Critical Power Test and a 20-min Functional Threshold Power Test in Cycling

**DOI:** 10.3389/fphys.2020.613151

**Published:** 2021-01-22

**Authors:** Bettina Karsten, Luca Petrigna, Andreas Klose, Antonino Bianco, Nathan Townsend, Christoph Triska

**Affiliations:** ^1^European University of Applied Sciences (EUFH), Berlin, Germany; ^2^Sport and Exercise Sciences Research Unit, University of Palermo, Palermo, Italy; ^3^Institut für Sportwissenschaft, Westfälische Wilhelms-Universität Münster, Münster, Germany; ^4^College of Health and Life Sciences, Hamad Bin Khalifa University, Doha, Qatar; ^5^Institute of Sport Science, Centre for Sport Science and University Sports, University of Vienna, Vienna, Austria; ^6^Leistungssport Austria, High Performance Unit, Brunn am Gebirge, Austria

**Keywords:** power-duration relationship, exercise tolerance, fatigue threshold, cycling performance, functional threshold power

## Abstract

To investigate the agreement between critical power (CP) and functional threshold power (FTP), 17 trained cyclists and triathletes (mean ± SD: age 31 ± 9 years, body mass 80 ± 10 kg, maximal aerobic power 350 ± 56 W, peak oxygen consumption 51 ± 10 mL⋅min^–1^⋅kg^–1^) performed a maximal incremental ramp test, a single-visit CP test and a 20-min time trial (TT) test in randomized order on three different days. CP was determined using a time-trial (TT) protocol of three durations (12, 7, and 3 min) interspersed by 30 min passive rest. FTP was calculated as 95% of 20-min mean power achieved during the TT. Differences between means were examined using magnitude-based inferences and a paired-samples *t*-test. Effect sizes are reported as Cohen’s *d*. Agreement between CP and FTP was assessed using the 95% limits of agreement (LoA) method and Pearson correlation coefficient. There was a 91.7% probability that CP (256 ± 50 W) was higher than FTP (249 ± 44 W). Indeed, CP was significantly higher compared to FTP (*P* = 0.041) which was associated with a trivial effect size (*d* = 0.04). The mean bias between CP and FTP was 7 ± 13 W and LoA were −19 to 33 W. Even though strong correlations exist between CP and FTP (*r* = 0.969; *P* < 0.001), the chance of meaningful differences in terms of performance (1% smallest worthwhile change), were greater than 90%. With relatively large ranges for LoA between variables, these values generally should not be used interchangeably. Caution should consequently be exercised when choosing between FTP and CP for the purposes of performance analysis.

## Introduction

Sport scientists, athletes, and coaches intuitively understand that as exercise intensity increases, a point is reached where a maximal metabolic steady state occurs, beyond which perceptions of effort and physiological perturbations progress more rapidly (for review see: Jones et al., 2019). These perceptions of physical discomfort are associated with mechanisms of peripheral fatigue which ultimately lead to task failure (Hureau et al., 2018). During laboratory testing this threshold is usually identified using lactate landmarks (e.g., lactate turning point, maximal lactate steady state or ventilatory thresholds). These thresholds are protocol dependent and may not align with a maximal oxidative steady state during constant load exercise, which has thus led to controversy (Jamnick et al., 2018; Iannetta et al., 2019; Poole et al., 2020). Conventionally, the maximal lactate steady state (MLSS) was believed to reflect a “true” maximal metabolic steady state (MMSS), however, it was recently demonstrated that an oxidative steady state can be maintained despite gradually increasing blood lactate (Brauer and Smekal, 2020). Furthermore, mathematical modeling of lactate kinetics suggests that a true equilibrium between maximal whole body lactate production and oxidation results in a gradually increasing blood lactate concentration (Beneke, 2003). Therefore, since the so-called critical power (CP) has been shown to lie within the intensity region which distinguishes steady state from non-steady state oxidative metabolism (Poole et al., 1988; de Lucas et al., 2013; Vanhatalo et al., 2016), an emerging consensus recognizes CP to more accurately represent a MMSS than the MLSS (Jones et al., 2019; Galan-Rioja et al., 2020).

Over the past 40 years, the CP concept has been studied extensively within the scientific literature and it has emerged as a simple mathematical model which not only describes the relationship between sustainable power and the development of fatigue during high intensity exercise, but which also provides an estimate of the maximal sustainable metabolic rate (Poole et al., 2016; Galan-Rioja et al., 2020). Nevertheless, there are some methodological considerations which might affect an accurate determination of CP (e.g., day-to-day variability, protocol, methodology, mathematical modeling) (Muniz-Pumares et al., 2019). The work rate at CP is closely associated with performance in endurance events (Kranenburg and Smith, 1996; Florence and Weir, 1997; Joyner and Coyle, 2008; Nimmerichter et al., 2017), and moreover, training above or below this MMSS leads to differences in physiological adaptations and specific performance outcomes (Vanhatalo et al., 2011; Iannetta et al., 2018). Due to the increasing availability of affordable on-the-bike power meters though, field-based methods of threshold assessment have been validated (Karsten et al., 2015; Triska et al., 2015). Additionally, the use of specialized and expensive laboratory equipment is not always justified and it requires technical expertise.

More recently, practical methods of threshold assessments have emerged such as field-based CP testing (Karsten et al., 2015; Triska et al., 2015; Muniz-Pumares et al., 2019), and the Functional Threshold Power (FTP). The FTP is defined as “the highest power that a rider can maintain in a quasi-steady state without fatiguing for approximately 1 hour” (Allen and Coggan, 2010), and has become widely popular amongst recreational and competitive cyclists for the purpose of aerobic capacity assessment and training prescription. However, to-date there is controversy as to whether FTP is related to CP or parameters of other threshold concepts (e.g., lactate landmarks or ventilatory thresholds) (Denham et al., 2017; Borszcz et al., 2018; Valenzuela et al., 2018; Sorensen et al., 2019). For example, Valenzuela et al. (2018) found non-significant differences between FTP and the second anaerobic threshold (AnT2) using the D_max_ method. Using measures of gas exchange, Denham et al. (2017) found the relative maximal oxygen uptake ($\dot{V}$O_2max_) to be significantly correlated with FTP. Also, Borszcz et al. (2018) assessed the agreement between FTP using a 20 min time trial (TT) and a 60 min TT, and the PO associated with the individual anaerobic threshold (IAnT; defined as a 1.5 mmol⋅L^–1^ increase above the point of a minimum ratio between blood lactate concentration and work rate). The authors concluded that despite strong correlations, the limits of agreement between the FTP estimates and IAnT were too wide to be used interchangeably. A major limitation of some of these aforementioned studies is that FTP was compared to parameters obtained from incremental exercise, which are known to be protocol dependent and may not align with indices like CP or MLSS obtained during sustained constant work rate exercise (e.g., Faude et al., 2009; Jamnick et al., 2018).

Critical power was originally defined as an exercise intensity that could be sustained for a “very long time” (Monod and Scherrer, 1965). CP can be determined using maximal self-paced TT efforts. These have been, when compared to the traditional constant power time-to-exhaustion approach, shown to be valid and reliable (Galbraith et al., 2014; Triska et al., 2017, 2020; Karsten et al., 2018). Only a limited number of published studies exist in the scientific literature which examine the relationship between CP derived from different protocols and FTP (MacInnis et al., 2018; Morgan et al., 2019). Therefore, the aim of the present laboratory-based study was to compare CP as an index of MMSS with FTP. We chose a test protocol for CP assessment that is not different from the traditional constant-work rate approach (Triska et al., 2017; Karsten et al., 2018) and also a widely used and recommended 20 min TT for FTP (Denham et al., 2017; Borszcz et al., 2018; Valenzuela et al., 2018), in a cohort of moderately trained cyclists.

## Materials and Methods

### Participants

Participants in this laboratory-based study were 17 moderately trained cyclists and triathletes (mean ± SD: age 31 ± 9 years, body mass 80 ± 10 kg, maximal aerobic power [MAP] 350 ± 56 W, peak oxygen consumption [$\dot{\text{V}}$O_2peak_]; 51 ± 10 mL⋅min^–1^⋅kg^–1^). All procedures performed were in accordance with the ethical standards of the institutional and/or national research committee and with the 1964 Helsinki declaration and its later amendments. Informed consent was obtained from all participants after information of the nature and any risks associated with this study were provided.

### Experimental Design

During visit one, $\dot{V}$O_2peak_ and MAP were determined during an incremental test. During visit two and three, participants performed either a CP or FTP test in randomized order. All subjects had previous experience at conducting TTs, and they were instructed to give a maximum effort for each test. Participants refrained from heavy exercise in the 24 h prior to testing, and food and caffeine for 3 h prior to testing. For all three visits participants were instructed to arrive at the laboratory in a fully rested and hydrated state. All testing was conducted on a Cyclus2 ergometer (RBM Electronics, Leipzig, Germany), which enables the participant to use their own personal racing bicycle.

### Peak Oxygen Uptake Test

After a standardized warm-up at 150 W and for 5 min, participants completed an incremental step test until volitional exhaustion. After 3-min baseline at ∼80 W, the test commenced at an intensity of 100 W with a step-like increase of 20 W min^–1^. Participants self-selected cadence throughout, and when this decreased by more than 10 rev⋅min^–1^ for 10 s despite strong verbal encouragement, the test was terminated. Pulmonary gas exchange was measured breath-by-breath using a Cortex MetaLyzer 3B gas analyzer (Cortex Biophysik, Leipzig, Germany), and heart rate (HR) was monitored via the ergometer. Outliers were excluded from further analysis after visual inspection by two independent researchers. MAP was calculated using the following equation:

(1) $$\text{MAP}={\text{P}}_{\text{L}}+\left(\text{t}/60\times {\text{P}}_{\text{I}}\right)$$

where P_L_ represents the last completed stage (W), t is time for the incomplete stage (s) and P_I_ is the step increment (W). $\dot{V}$O_2peak_ was taken as the highest 30-s rolling-average during the incremental test. The mean ± SD duration of the peak oxygen test was 13.5 ± 2.8 min.

### Critical Power Testing

Critical power was determined using maximal self-paced TT efforts over the durations of 12, 7, and 3 min with a 30 min passive rest between efforts. The protocol started with a 5 min warm-up phase at 100 W immediately followed by a switch of the ergometer into TT mode, where resistance increases or decreases as a function of cadence and pedal force. During the TT, participants were allowed to self-pace via use of a virtual gear changer mounted to the handlebars. Feedback of elapsed time and strong encouragement was provided throughout and participants were asked to produce the highest average PO possible. Heart rate (HR) was measured continuously and rate of perceived exertion (RPE) was recorded immediately at the end of trials. HR within 10 beats of age-predicted HR maximum and RPE values above 18 were taken as an indicator for a maximal effort and accepted as a successful test.

Critical power and its related maximum work above CP (*W′*) were determined using two linear and one hyperbolic model:

$$\mathrm{linear}\mathrm{work}\mathrm{vs}.\mathrm{time}:W_{\text{lim}}=W^{\prime }+\mathrm{CP}\times \text{t}$$

(2) (Moritani et al., 1981)

$$\mathrm{linear}\mathrm{PO}\mathrm{vs}.\mathrm{inverse}\mathrm{of}\mathrm{time}:\mathrm{PO}=W^{\prime }\times \left(1/t\right)$$

(3) +CP⁢(Whipp et al., 1982)

$$\mathrm{hyperbolic}\mathrm{PO}\mathrm{vs}.\mathrm{time}:t=W^{\prime }/\left(\mathrm{PO}-\mathrm{CP}\right)$$

(4) (Hill, 1993)

The inverse time linear model (*P* = W*′*/t + CP) provided the lowest combined standard error of the estimate (SEE) (i.e., sum of SEE% of CP and *W′*) and was consequently used. The linear PO vs. inverse of time model provided the lowest combined SEE for all participants (*n* = 17) and was therefore used for further analysis. This model was also used to calculate predicted maximal 20 min PO (p20_MMP_) values (e.g., 15000 × (1/1200) + 300).

### Functional Threshold Power Testing

Functional threshold power was estimated from a single 20 min TT effort similar to recent research (Denham et al., 2017; Borszcz et al., 2018; Valenzuela et al., 2018) and described elsewhere (Allen and Coggan, 2010). For consistency with the CP protocol, this TT also commenced with a 5 min warm-up at 100 W. Throughout the 20 min TT, participants were allowed to self-pace, and elapsed time feedback was provided as per CP testing. HR was measured continuously and RPE was recorded immediately at the end of trials. HR within 10 beats of age-predicted HR maximum and RPE values above 18 were taken as an indicator for a maximal effort and accepted as a successful test. FTP was calculated as 95% of the 20 min maximal measured PO (20_MMP_) obtained during the TT (Allen and Coggan, 2010; Denham et al., 2017; Borszcz et al., 2018; Valenzuela et al., 2018).

### Statistics

Data were first examined for normality using the Shapiro-Wilk test. Differences between means were assessed using magnitude based inferences (Batterham and Hopkins, 2006) where the “smallest worthwhile change” in PO considered to be meaningful in terms of practical significance, was set at 1% of the mean CP estimate using an Microsoft Excel spreadsheet (Paton and Hopkins, 2001; Batterham and Hopkins, 2006). The agreement between variables was assessed using 95% limits of agreement (LoA) (Bland and Altman, 1986) using GraphPad Prism (version 6.00 for Mac; GraphPad Software, La Jolla CA, www.graphpad.com). Pearson product moment correlation was used to provide an estimate of strength of association between variables, and linear regression was used to calculate the SEE associated with prediction of 20_MMP_ and FTP from CP, and also p20_MMP_ and pFTP, respectively. Lin’s concordance coefficient and the intraclass correlation coefficient (ICC) was used to evaluate the agreement between methods assessed by a Microsoft Excel spreadsheet. To assess differences between CP, FTP, and 20_MMP_ a repeated-measures analysis of variance was used. Partial squared eta ($\eta_{p}^{2}$) was used to calculate for effect sizes (small $\eta_{p}^{2}$ = 0.02, moderate $\eta_{p}^{2}$ = 0.13, and large $\eta_{p}^{2}$ = 0.26). Effect sizes of the *post hoc* tests are reported as Cohen’s *d* calculated as the quotient of mean differences and variance (small *d* = 0.2; moderate *d* = 0.5; large *d* = 0.8). The typical error of the estimate and the coefficient of variation (%) (Hopkins, 2000) were used to assess validity, where CP was taken as the criterion variable and FTP as practical variable assessed by an Microsoft Excel spreadsheet. Statistical significance was set at *P* < 0.05 and raw data is reported as mean ± SD. All analyses were conducted using SPSS statistical software package 27 (IBM SPSS statistics, SPSS Inc., Chicago, United States) unless stated otherwise.

## Results

**FIGURE 1:**
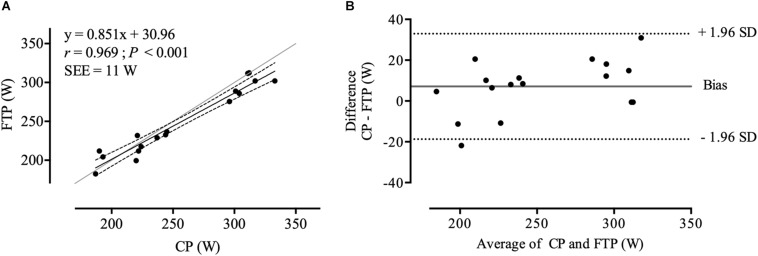
**(A)** correlation between CP and FTP. The gray line represents the line of identity, the black line shows the line of best fit, and the dotted lines are the ± 95% confidence intervals. **(B)** Bland-Altman plots of CP/FTP. The gray horizontal line represents the mean bias between values, and the dotted line represents the 95% LoA.

All data were normally distributed (*P* > 0.05). Descriptive data is presented in Table 1 and individual CP and *W′* results from linear and hyperbolic models including combined SEE are depicted in Table 2. Significant main effects and a *large* effect size were found between CP, FTP, and 20_MMP_ (*F*_2,32_ = 13.029; *P* < 0.001; $\eta_{p}^{2}$ = 0.45). Bonferroni *post hoc* procedures revealed significant differences and *large* effect sizes only between FTP and 20_MMP_ (*P* < 0.001; *d* = 2.44), but not between CP and FTP (*P* = 0.122; *d* = 0.04), and between CP and 20_MMP_ (*P* = 0.200; *d* = 0.04). CP and FTP were significantly correlated (*r* = 0.969; *P* < 0.001) (Figure 1A). There was a 91.7% probability that CP was higher than FTP (likely). The mean bias and the 95% LoA between CP and FTP were 7 ± 13 W (95% LoA: −19 to 33 W) (Figure 1B). The Lin’s concordance coefficient between CP and FTP was *r* = 0.950 (95% confidence limits, [CL]: 0.877 to 0.980), the ICC was *r* = 0.976 (95% CL: 0.915 to 0.991) and the typical error in raw unit was 13 W (95% CL: 9 to 20 W) and expressed as a coefficient of variation (%) was 5.6% (95% CL: 4.1 to 8.8%). Mean peak HR and mean RPE values for the 20 min TT, 12 min TT, 7 and 3 min TT were as follows: 184 ± 12 b min^–1^ and 19 ± 1; 184 ± 13 b min^–1^ and 19 ± 1; 181 b min^–1^ ± 12 b min^–1^ and 19 ± 1; 178 ± 14 b min^–1^ and 19 ± 1 (*P* > 0.05).

**TABLE 1:** Performance measures obtained from the tests (mean ± SD).

**Measure**	**Group (*n* = 17)**
CP (W)	25650
CP SEE (W)	55
CP SEE (%)	2.93.0
*W′* (kJ)	16.976.907
CP SEE (kJ)	1.91.5
CP SEE (%)	13.816.5
20_MMP_ (W)	26246
p20_MMP_ (W)	27051
FTP (W)	24944
pFTP (W)	26150

*CP, critical power; SEE, standard error of the estimate; *W′*, maximal work accomplished above CP; *R*^2^, coefficient of determination; 20_*MMP*_, 20 min maximal mean power; p20_*MMP*_, predicted 20 min maximal mean power; FTP, functional threshold power; pFTP, predicted functional threshold power.*

**TABLE 2:** Individual CP and *W′* for the linear models (power vs. 1/time and work vs. time) and the hyperbolic model.

**No.**	**linear model (power vs.-1/time)**			**linear model (work vs. time)**			**hyperbolic model**		
	**CP**	***W′***	**combined SEE**	**CP**	***W′***	**combined SEE**	**CP**	***W′***	**combined SEE**
1	224	13.850	11.9%	228	12.559	20.8%	232	9.679	28.4%
2	222	11.574	18.1%	226	9.891	33.7%	233	5.435	56.4%
3	312	21.347	4.4%	314	20.632	7.1%	316	19.240	8.4%
4	245	8.614	32.3%	238	10.965	42.7%	233	14.027	37.8%
5	317	13.312	3.7%	316	13.715	5.8%	315	14.419	6.2%
6	237	16.467	8.3%	234	17.516	12.5%	231	19.255	12.8%
7	190	23.930	8.2%	194	22.639	12.8%	198	20.016	15.3%
8	301	17.012	11.9%	296	18.614	17.7%	292	21.161	17.6%
9	296	12.125	11.1%	293	13.243	16.8%	290	15.026	16.8%
10	311	24.481	8.3%	307	25.991	12.4%	303	28.502	12.7%
11	193	20.300	23.8%	185	23.642	32.4%	176	28.515	30.5%
12	244	24.146	10.6%	239	25.955	15.5%	234	28.909	15.6%
13	304	26.966	14.1%	297	29.721	20.3%	289	34.055	19.9%
14	220	3.358	24.7%	222	2.609	53.5%	220	3.420	177.5%
15	187	9.480	71.2%	171	15.911	87.5%	158	22.094	84.1%
16	221	26.289	6.3%	218	27.395	9.2%	215	29.290	9.5%
17	333	15.301	1.5%	333	15.486	2.4%	332	15.817	2.6%
mean	256	16.974	15.9%	254	18.028	23.7%	251	19.345	32.5%
SD	50	6.907	16.4%	51	7.276	21.3%	52	8.697	42.6%

*CP, critical power; *W′*, maximal work accomplished above CP; SEE, standard error of the estimate.*

## Discussion

The primary finding of this study was that for moderately trained cyclist, mean CP was non-significantly higher than FTP (i.e., 95% of 20_MMP_). The probability of a meaningful difference was >90%, however, the effect size was only of a *trivial* order. The results also revealed wide LoA between CP and FTP (± 24 W), a large bias (−12 W) and large intraindividual variations (± 12 W). Nevertheless, the variables were strongly correlated (*r* = 0.969). According to Cohen, the typical error of the estimate between CP and FTP can be interpreted as small (13 W; 5.6%). This, however, is arguably above the 5%, which is the commonly accepted upper limit in sport science research (Hopkins, 2000). Our results are supported by Borszcz et al. (2018) who reported notably larger LoA between the IAnT and FTP and 20_MMP_, respectively, than reported in the present study. Furthermore, our results are consistent with Valenzuela et al. (2018), who identified poor LoA and a significant difference between FTP and the AnT2 in moderately trained athletes. Interestingly, however, LoA between FTP and the AnT2 were stronger and non-significantly different in well trained athletes (Valenzuela et al., 2018).

We observed a mean difference of 7 ± 13 W between CP and FTP. Given that a 1% difference in PO (∼2.6 W in our study) is considered a “smallest worthwhile change,” the disparity between CP and FTP equates to a high probability, that a meaningful difference exists in practice (Paton and Hopkins, 2001, 2006; Batterham and Hopkins, 2006). Even though using different methods, Borszcz et al. (2018) also demonstrated wide LoA between the IAnT and FTP determined from a 20 min TT (−62 to 60 W). Notably in this study is the difference of 5 W (2%) between measured 20_MMP_ (231 W) and predicted 20 min PO (236 W). This might raise questions about the application rule of 5% from the 20 min FTP values as postulated by Allen and Coggan (2010). Although Valenzuela et al. (2018) did not find significant differences and a trivial effect size between FTP and the AnT2, their results also demonstrated wide LoA. Arguably, these findings question the underlying physiology of the FTP concept. With respect to the present study it is noteworthy that Borszcz et al. (2018) and Valenzuela et al. (2018) did not use CP as criterion. According to the correlation between CP and FTP only 6.1% of the variance between CP and FTP (*R*^2^ = 0.939) are explained by other factors. Nevertheless, these 6.1% are suggested to notably question the interchangeable use of CP and FTP. Collectively, these findings do not support the assertion by Allen and Coggan (2010) that FTP always corresponds to the highest the PO maintainable in a quasi-steady state. Importantly, the identified differences and error between CP and FTP values in the present study suggest that these estimates of threshold power should not be used interchangeably.

Interestingly, there were no significant differences, apart from a *trivial* effect size, between CP and 20_MMP_. However, previous research has shown that time-to-fatigue at CP equals ∼23 min in both untrained (Poole et al., 1988) and trained cyclists (de Lucas et al., 2013). Conversely, CP has been found to reside ∼20 W above MLSS intensity (Pringle and Jones, 2002) which indicates a clear difference between CP and MLSS (Jones et al., 2019; Galan-Rioja et al., 2020). It is, however, noteworthy that other authors found small differences (∼1 W) between CP and MLSS (Keir et al., 2015). These different results are suggested to be due to the fact that both CP and MLSS are protocol depended which is a clear limitation. We therefore suggest that CP tends to reside above MLSS. Therefore, a 20 min TT would more closely align with CP and 95% of a 20 min TT would more closely align with MLSS where time-to-fatigue is 55 ± 8.5 min (Baron et al., 2008). This is also confirmed by the suggestions of Jones et al. (2019) that MLSS and CP cannot be used interchangeably as a boundary between the heavy and the severe intensity exercise domain. As a result of these findings, it is suggested that FTP (calculated as 95% of 20_MMP_) is generally lower than CP.

The difference in CP and FTP reported here raises another important question, that of which estimate of threshold power more closely aligns with the underlying physiological determinants. A recent definition of CP is as follows: “In contrast to historical definitions, CP is now considered to represent the greatest metabolic rate that results in wholly-oxidative energy provision” (Poole et al., 2016). If the ATP demand is not supplied by “wholly-oxidative” metabolism, the additional anaerobic contribution leads to a decline in [PCr] and increasing $\dot{V}$O_2_ uptake (Korzeniewski and Rossiter, 2015). Thus, the most direct non-invasive method of validation is to measure *in vivo* muscle metabolism slightly above and below the estimate of threshold. To-date, only one study has conducted these measurements using ^31^P magnetic resonance spectroscopy during single-leg knee extension exercise (Jones et al., 2008). These authors reported a progressive loss of [PCr], [P_i_], and [H^+^] homeostasis at work rate ∼10% above CP, but attainment of a steady state when the work rate was 10% below CP. The most direct non-invasive method of physiological validation for whole-body exercise is to measure $\dot{V}$O_2_ uptake. Several studies have reported the occurrence of a $\dot{V}$O_2_ steady state corresponding to a work rate at, or slightly below CP, whereas non-steady state $\dot{V}$O_2_ were observed slightly above CP (Poole et al., 1988; de Lucas et al., 2013; Murgatroyd et al., 2014; Vanhatalo et al., 2016). In each case, the limit of tolerance was reached markedly sooner at the work rate slightly above CP. Collectively, these studies provide evidence that estimates of CP correspond to an intensity which demarcates steady state from non-steady state oxidative metabolism. It should be noted, however, that estimates of CP are protocol dependent (e.g., Hill and Smith, 1994; Bishop et al., 1998; Mattioni Maturana et al., 2018; Triska et al., 2018), thus only those protocols which have been physiologically validated should be considered to represent threshold intensity. In the current study we used a protocol that has been shown to derive CP and *W′* estimates with a low SEE and thus a high accuracy (Triska et al., 2017; Karsten et al., 2018).

One of the differences between CP and FTP is that the concept of CP also incorporates *W′*. During the TT used for the determination of CP, energy contribution is not “wholly-oxidative,” but also includes an energy contribution derived from expenditure of *W′*. For example, in the present study, mean *W′* was 16.6 kJ which equates to an additional 4.6 W (averaged over 60 min) above the maximal PO estimated to be “wholly-oxidative.” Since anaerobic energy provision contributes to *W′* (Poole et al., 2016), then a valid determination of a threshold using a single maximum effort (such as FTP), requires that either no expenditure of *W′* occurs (PO at or below CP), or if expenditure of *W′* occurs, then due to some other mechanism a corresponding decrease in average power must also occur. This has to be done to offset the additional energy contribution arising from expenditure of *W′*. In fact, it should be expected that predicted MMP over a given duration i.e.: *P* = *W′* (1/t) + CP, where *t* = the duration of the task, should be close to the actual performance power. Further analysis revealed that p20_MMP_ and 20_MMP_ were significantly different (mean difference: 8 ± 10 W; *P* = 0.005; *d* = 0.07). The discrepancy in mean values here is potentially explained by emergence of fatigue mechanisms that are not dominant during the short duration TTs used to estimate CP. For example, central fatigue has been shown to increase during a 20 and 40 km TTs compared to a 4 km TT (Thomas et al., 2015), and also during a time to exhaustion task lasting ≈11 min as compared to one lasting ≈3 min (Thomas et al., 2016), thus for TTs lasting even only 20 min, actual sustainable power may diverge from linearity as predicted by the 2-parameter CP model. Also, cycling efficiency at a 60% maximum minute PO intensity has been found to decrease during 2 h cycling below CP (Hopker et al., 2017), and W′ has been demonstrated to markedly decrease with glycogen depletion (Miura et al., 2000). Thus, it may be possible for both CP and *W′* to decline during prolonged higher intensity endurance exercise (i.e., short duration TTs), which suggests the domain of validity of the 2-parameter CP model should be limited to durations less than 20 min.

There are certain key limitations to the present study. In the absence of pulmonary gas exchange and measurements slightly above and below both CP and FTP, we were unable to validate these estimates according to the physiological criteria which best describes the threshold phenomenon. Thus, further studies are required to establish the physiological validity of the FTP concept. Moreover, the prediction of performance PO beyond 20 min based on the CP model can be questioned. This is a limitation of the power-duration relationship modeling procedure. Furthermore, we did not conduct familiarization trials but used only experienced cyclists or triathletes. Finally, it is currently unclear if different warm-up protocols influence FTP and this should consequently be addressed in further research.

The “smallest worthwhile change” in PO during a sustained TT which approximates threshold intensity is approximately 1% in elite athletes (Paton and Hopkins, 2006). In the present study, such a difference equates to a ∼92% chance that CP estimated from the 2-parameter inverse-time model is greater than FTP estimated via the 95% of 20_MMP_ method. Furthermore, the LoA between CP and FTP were wide and therefore, these estimates of threshold intensity should not be used interchangeably.

## Data Availability Statement

The raw data supporting the conclusions of this article will be made available by the authors, without undue reservation.

## Ethics Statement

The studies involving human participants were reviewed and approved by the University of Greenwich. The patients/participants provided their written informed consent to participate in this study.

## Author Contributions

BK conceptualized the study, conducted testing, and wrote parts of manuscript. LP and AB wrote parts of the manuscripts. AK conducted testing and wrote parts of the manuscripts. NT conducted statistical analysis and wrote parts of the manuscript. CT analyzed data and wrote parts of the manuscript. All authors contributed to the article and approved the submitted version.

## Conflict of Interest

The authors declare that the research was conducted in the absence of any commercial or financial relationships that could be construed as a potential conflict of interest.
